# The Polycomb Group Protein EED Is Dispensable for the Initiation of Random X-Chromosome Inactivation

**DOI:** 10.1371/journal.pgen.0020066

**Published:** 2006-05-05

**Authors:** Sundeep Kalantry, Terry Magnuson

**Affiliations:** Department of Genetics and the Carolina Center for the Genome Sciences, University of North Carolina at Chapel Hill, Chapel Hill, North Carolina, United States of America; The Babraham Institute, United Kingdom

## Abstract

The Polycomb group (PcG) proteins are thought to silence gene expression by modifying chromatin. The Polycomb repressive complex 2 (PRC2) plays an essential role in mammalian X-chromosome inactivation (XCI), a model system to investigate heritable gene silencing. In the mouse, two different forms of XCI occur. In the preimplantation embryo, all cells undergo imprinted inactivation of the paternal X-chromosome (Xp). During the peri-implantation period, cells destined to give rise to the embryo proper erase the imprint and randomly inactivate either the maternal X-chromosome or the Xp; extraembryonic cells, on the other hand, maintain imprinted XCI of the Xp. PRC2 proteins are enriched on the inactive-X during early stages of both imprinted and random XCI. It is therefore thought that PRC2 contributes to the initiation of XCI. Mouse embryos lacking the essential PRC2 component EED harbor defects in the maintenance of imprinted XCI in differentiating trophoblast cells. Assessment of PRC2 requirement in the initiation of XCI, however, has been hindered by the presence of maternally derived proteins in the early embryo. Here we show that *Eed* ^−*/*−^ embryos initiate and maintain random XCI despite lacking any functional EED protein prior to the initiation of random XCI. Thus, despite being enriched on the inactive X-chromosome, PcGs appear to be dispensable for the initiation and maintenance of random XCI. These results highlight the lineage- and differentiation state–specific requirements for PcGs in XCI and argue against PcG function in the formation of the facultative heterochromatin of the inactive X-chromosome.

## Synopsis

During the development of an embryo, an equal population of cells gives rise to different tissues and organs and this occurs via the activation and silencing of different sets of genes. The Polycomb group (PcG) proteins are required for silencing genes for proper development. PcGs play an important role in silencing of one of the two X-chromosomes in female mammals. XX females inactivate one X-chromosome to equalize X-linked gene dosage to that of XY males. X-chromosome inactivation (XCI) is established in cells of the early embryo and when these cells divide they maintain silencing of the same X-chromosome. XCI, therefore, is a model system to understand long-term gene regulation. Here the authors test whether PcGs help initiate XCI by examining mouse embryos lacking the essential PcG protein EED. Their results show that XCI in embryonic cells occurs normally despite lacking EED. PcGs therefore are not required to initiate or maintain XCI in embryonic cells. The findings shed light on the specific requirements for PcGs in XCI and argue against a general silencing function for PcGs in XCI.

## Introduction

Eukaryotic gene expression is regulated by a coordinated interplay of chromatin-remodeling factors, histone-modifying enzymes, general and gene-specific transcription factors, and RNA polymerases. The chromatin remodeling machinery and histone modifiers work further to impart information for the maintenance of gene expression states through cell division. This form of epigenetic control of gene regulation is correlated with covalent modifications of histones, such as acetylation, methylation, and phosphorylation. These modifications are hypothesized to recruit specific protein complexes or to affect nucleosome structure, which in turn directly or indirectly influence the activity of the basal transcription machinery [1,2].

The Polycomb group (PcG) is a large and evolutionarily conserved set of genes whose products act in multimeric complexes to modify histones, which are then thought to cause stable and heritable states of transcriptional repression [3,4]. The Polycomb repressive complex 2 (PRC2) methylates lysine 27 of histone H3 (H3-K27) both in vivo and in vitro [5–9]. H3-K27 methylation in turn provides a substrate for the assembly of Polycomb repressive complex 1 (PRC1) via binding of the Polycomb (Pc) protein [5,8,10]. PRC1 is thought to compact chromatin in a transcriptionally repressed state [5,8,11]. The core components of *Drosophila* PRC1 have been shown to physically compact nucleosomal arrays and inhibit chromatin remodeling and transcription in vitro [12,13].

X-chromosome inactivation (XCI) is the process by which male and female mammals achieve dosage equality of X-linked genes by transcriptional silencing of one of the two X-chromosomes in females [14]. In mice two distinct forms of XCI exist, termed imprinted and random X-inactivation [14–17]. In the four-cell embryo, all cells initiate imprinted inactivation of the paternal X-chromosome (Xp) [18–20]. Then, at the late blastocyst stage, this imprint is erased only in the cells destined to give rise to the embryo proper (inner cell mass [ICM]) [18,19]. These cells then randomly inactivate either the maternal or the paternal X-chromosome. Cells of the two other lineages at this stage (the trophectoderm and primitive endoderm, both extraembryonic), on the other hand, maintain imprinted XCI of the Xp. The same X-chromosome remains inactive in all descendants of progenitor cells in which it is first silenced [21]; XCI therefore provides a model system to investigate heritable forms of transcriptional regulation.

The PRC2 complex has been implicated in the initiation phase of XCI. In mouse embryos, XCI is preceded by the transcription of the X-linked nonprotein coding *X (inactive)-specific transcript (Xist)* from the Xp [18–20,22]. The *Xist* RNA begins to coat the Xp at the four-cell stage and spreads progressively over several cleavage divisions, culminating in complete coating of the Xp at the blastocyst stage [22]. The spread of *Xist* coating correlates with the gradual transcriptional silencing of genes along the Xp [22]. In the eight-cell embryo, the *Xist* coated region of the Xp is found devoid of the histone modifications dimethyl lysine 4 of histone H3 and acetylated histone H3, both of which are associated with actively transcribed chromatin [18]. Subsequently, at the morula stage, PRC2 proteins and trimethyl lysine 27 of histone H3 (H3-3mK27) are found enriched on the Xp [18,19].

The timing of epigenetic modifications that characterize the inactive-X (Xi) during random XCI closely parallels that during imprinted XCI [23]. The core PRC2 components, EED, EZH2, and SUZ12, as well as the histone modification they mediate, H3-3mK27, are found enriched on the Xi early in random XCI, concomitant with or soon after *Xist* RNA coating of the Xi [19,24–27]. EED and H3-3mK27 have been shown to accumulate on the Xi when random XCI initiates in epiblast cells of post–implantation stage embryos [19]. Embryonic stem (ES) cells, which are derived from the ICM of blastocyst stage embryos, have often been used to study the epigenetic events associated with random XCI [28]. Undifferentiated female ES cells harbor two active X-chromosomes, one of which becomes stochastically inactivated during differentiation [29]. Inactivation begins early in differentiation with the coating of the prospective Xi by *Xist,* followed closely by the accumulation of EED, EZH2, SUZ12, and H3-3mK27 [24–27]. This temporal pattern of Xi accumulation in the developing embryo as well as in differentiating ES cells has led to the suggestion that the PcG proteins are required to stably initiate random XCI [24,25].

We first suggested a role for PcGs in XCI when female embryos mutant for the mouse PcG gene *Embryonic Ectoderm Development (Eed^17Rn5-3354SB^)* were found to harbor defects in the maintenance of imprinted XCI, using an X-inactivated paternal X-linked *GFP* transgene to monitor defects in XCI [30]. We further demonstrated that the Xp reactivation does not occur in undifferentiated mutant cells of the trophectoderm lineage but instead is restricted to differentiating mutant trophoblast cells [31]. These results indicated that EED and PRC2 are required to maintain imprinted XCI by preventing the differentiation-induced reactivation of the Xi.

Although EED is found enriched on the Xi early in both imprinted and random XCI and is clearly necessary to stably maintain imprinted XCI, it is not known whether EED is required for initiation of XCI. Defining a role for EED in the initiation of XCI has been precluded thus far by the presence of maternal EED protein in the early embryo [18,19,24,25,32,33]. Whereas zygotic *Eed* transcription is first evident at embryonic day 5.5 (E5.5), EED protein is detected on the Xi at E3.5. *Eed* ^−*/*−^ embryos, therefore, harbor considerable amounts of maternally derived EED protein during the stages when imprinted XCI is initiated in the preimplantation embryo. Here we address whether PcGs initiate XCI by assessing if EED protein is present in *Eed* ^−*/*−^ embryos prior to the initiation of random XCI in peri-implantation stage embryos. We also address conclusively whether random XCI is affected in the absence of EED.

Our results show that there is no functional EED protein during the developmental window when random XCI initiates, and that despite the lack of EED protein both initiation and maintenance of random XCI occur normally in *Eed* ^−*/*−^ embryos.

## Results

Although zygotic expression of *Eed* is first detected at E5.5, EED protein is found abundantly in preimplantation embryos at least up to the late blastocyst stage (E3.5) [18,19,24,25,32,33]. This maternally derived pool of EED may help initiate imprinted XCI which occurs during preimplantation development, and persist long enough to initiate random XCI. Random XCI initiates following the imprint erasure event that occurs at the late blastocyst stage.

To define when maternally derived EED becomes depleted, we analyzed EED protein content by immunofluorescence (IF) in preimplantation and peri-implantation stage embryos generated from a cross of *Eed^+/−^* animals. One-fourth of the embryos generated from this cross are expected to be genotypically *Eed* ^−*/*−^ and lack all EED protein after depletion of maternal EED. The sire in this cross also carried on its X-chromosome a mutation in the *Tsix* gene (see below) and a *GFP* transgene. The X-linked *GFP* transgene is exclusively transmitted to female embryos and is expressed prior to XCI-mediated silencing [30,34]. This transgene thus facilitates the identification of female embryos and the analysis of paternal X-chromosome activity.

All female embryos examined at the early blastocyst stage (10 of 10) showed Xi enrichment of the EED protein in all cells (Figure 1A). Similarly, all embryos at the late blastocyst stage (i.e., those that had hatched from the zona pellucida; 12 of 12) also displayed clear Xi enrichment of EED (Figure 1B). At this stage, however, this Xi accumulation is evident only in the trophectoderm cells, which stably maintain imprinted XCI. The ICM is conspicuously devoid of EED enrichment on the Xi, indicating that it has undergone erasure of the imprint that ensures preferential Xp-inactivation. Notably, none of the 22 embryos examined at the blastocyst stages lacked EED protein.

**Figure 1:**
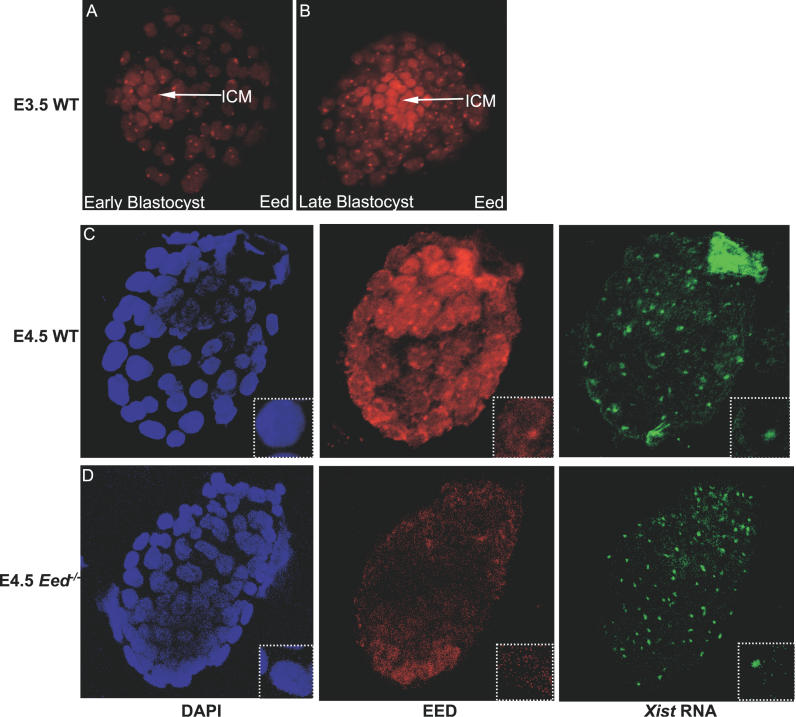
Absence of EED Protein in *Eed* ^−*/*−^ Embryos at E4.5 (A) IF detection of EED protein in an early E3.5 blastocyst stage embryo showing Xi enrichment of EED in all cells. (B) IF detection in a hatched, late-stage E3.5 blastocyst shows accumulation of EED on the Xi in trophoblast cells, an extraembryonic cell type that maintains imprinted XCI of the Xp but lacking Xi enrichment in ICM cells. The ICM cells at this stage of development undergo erasure of the imprint that ensures preferential transcriptional inactivation of the Xp. (C) IF detection of Xi-enriched EED protein in a WT E4.5 embryo. The Xi is coated with the *Xist* RNA, detected by FISH. DAPI staining detects nuclei. (D) IF-FISH staining of an E4.5 female embryo showing an absence of EED protein. The EED antibody staining seen is background and does not overlap with the Xi, as marked by *Xist* RNA coating.

We next analyzed E4.5 female embryos generated from the *Eed^+/^* ^−^ cross for the presence of EED protein. Of 14 embryos stained for EED, ten were positive for EED and showed accumulation on the Xi (marked by *Xist* RNA coating) (Figure 1C). Four E4.5 embryos, however, showed an absence of EED protein (Figure 1D).

We also assayed E4.5 embryos for the histone modification H3-3mK27. EED is required to catalyze the H3-3mK27 modification, as *Eed* ^−*/*−^ embryos as well as *Eed* ^−*/*−^ ES and TS (trophoblast stem) cells lack H3-3mK27 [9,24,31]. Whereas E4.5 embryos that accumulated EED on the Xi also always demonstrated H3-3mK27 Xi enrichment, absence of EED protein invariably resulted in lack of H3-3mK27 (Figure 2). Thus, maternally loaded EED protein and its activity in the form of H3-3mK27 appear to be depleted by E4.5.

**Figure 2:**
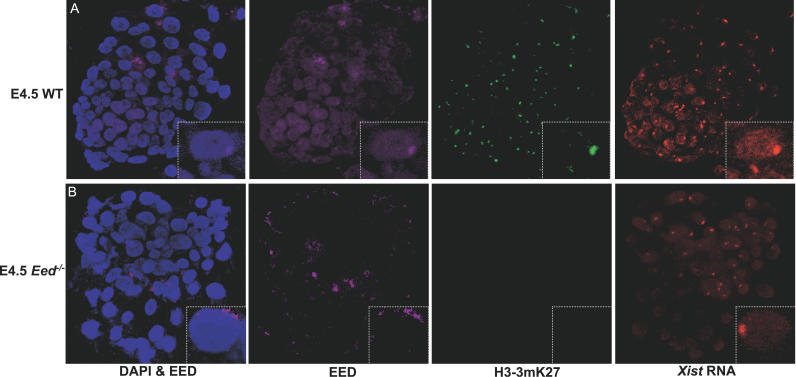
Absence of H3-3mK27 in *Eed* ^−*/*−^ Embryos at E4.5 (A) IF-FISH detection shows Xi enrichment of EED protein and H3-3mK27 in a WT E4.5 embryo. The Xi is marked by *Xist* RNA coating, detected by FISH. DAPI staining detects nuclei. (B) An E4.5 female embryo that is devoid of EED and H3-3mK27. The EED antibody staining seen is background and does not overlap with the Xi, as denoted by *Xist* RNA coating.

We then sought to determine if the absence of EED activity preceded the initiation of random XCI in the ICM lineage. The erasure of the Xp imprint in the ICM at the late blastocyst stage results in loss of the Xi enrichment of both EED and H3-3mK27. Their subsequent appearance on the Xi in the epiblast, which is ICM derived, at E5.5 coincides with the initiation of random XCI [19]. A majority of E4.5 embryos examined (17/22) showed clear Xi accumulation of EED or H3-3mK27 in the trophectodermal cells (Figure 3A and unpublished data). Cells of the epiblast, marked by the expression of the paternal X-linked *GFP* transgene *(Xp-GFP),* lack Xi enrichment of EED or H3-3mK27, indicating that these cells have yet to undergo random XCI (Figure 3A). *Xp-GFP* expression in the epiblast cells reflects the reactivation of the Xp due to imprint erasure. A proportion of E4.5 embryos (5 of 22), however, did not show noticeable EED or H3-3mK27 staining in any cell of the embryo, indicating that they are *Eed* ^−*/*−^ (Figure 3B and unpublished data).

**Figure 3:**
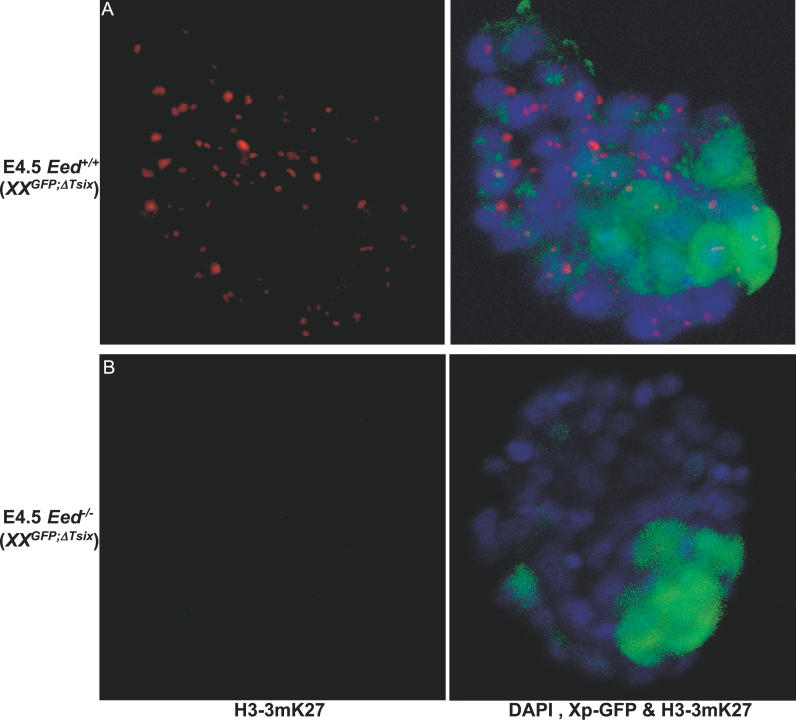
Absence of EED Activity prior to the Initiation of Random XCI in *Eed* ^−*/*−^ Embryos (A) IF detection of H3-3mK27 in a WT E4.5 embryo shows H3-3mK27 enrichment on the Xi in all trophectodermal cells, but which is largely absent in the ICM/epiblast cells indicating that these cells have not undergone random XCI. The selective expression of the paternal X-linked *GFP* transgene *(Xp-GFP)* in the ICM lineage indicates that these cells have erased the imprint that ensures imprinted XCI of the Xp. Whereas the ICM has erased the imprint, the trophectoderm has stably maintained imprinted XCI of the Xp, and hence is negative for *Xp-GFP*. DAPI staining detects the nuclei. (B) IF staining showing a lack of histone H3-3mK27 accumulation on the Xi in all cells at E4.5, indicating that EED activity is absent in mutant embryos prior to the initiation of random XCI.

The epiblast or epiblast derivatives of wild-type (WT) postimplantation embryos at E5.5, E6.5, and E7.5, however, all showed Xi accumulation of H3-3mK27 or EED and *Xist* RNA, signifying that random XCI has initiated in these cells (Figures 4 and 5 and unpublished data). A subset of female E5.5 embryos (4/18) derived from the *Eed^+/^* ^−^ cross lacked all H3-3mK27 staining and are classified as *Eed* ^−*/*−^ (Figure 4). Similarly, epiblast cells isolated from E6.5 *Eed* ^−*/*−^ embryos showed a complete absence of EED protein (Figure 5). The absence of EED and/or H3-3mK27 in E4.5-E6.5 embryos, therefore, indicates that maternal EED protein and its activity are depleted in *Eed* ^−*/*−^ embryos prior to the initiation of random XCI and are no longer detected during the developmental window when random XCI normally initiates.

**Figure 4:**
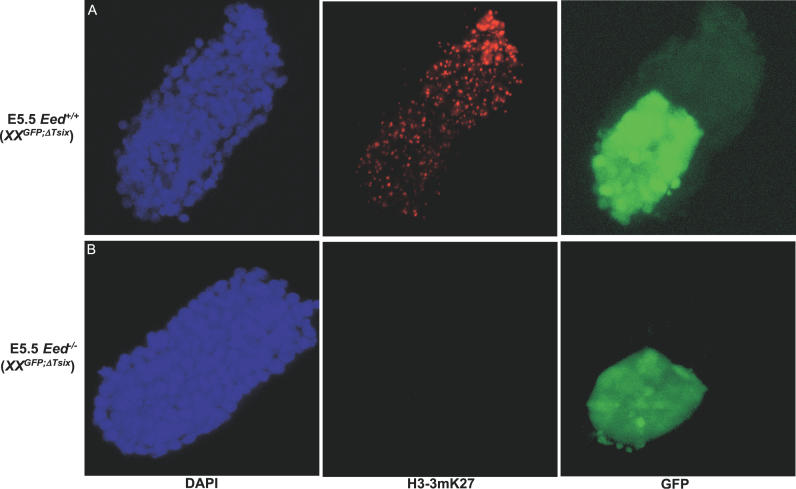
Enrichment of H3-3mK27 on the Xi during Initiation of Random XCI (A) IF detection in a WT E5.5 embryo showing Xi accumulation of H3-3mK27 in all cells of both the extraembryonic and embryonic lineages, i.e., the trophectoderm-derived extraembryonic ectoderm and the epiblast, which undergo imprinted and random XCI, respectively. (B) *Eed* ^−*/*−^ embryos lack H3-3mK27.

**Figure 5:**
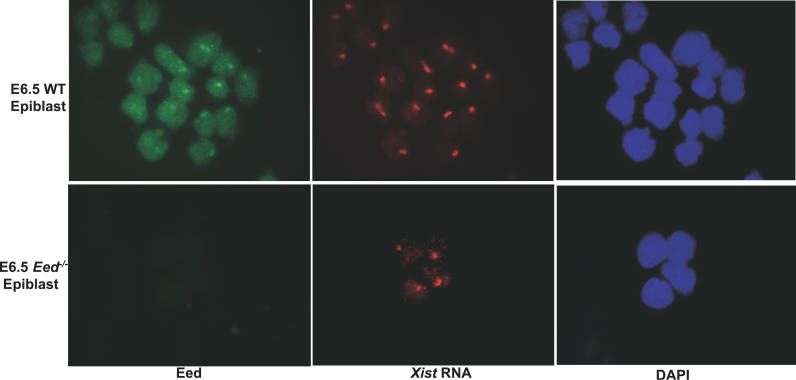
Absence of EED Protein in Epiblast Cells of E6.5 *Eed* ^−*/*−^ Embryos IF-FISH detection shows that whereas isolated E6.5 WT epiblast cells accumulate EED on the Xi, denoted by *Xist* RNA coating, epiblast cells from *Eed* ^−*/*−^ female embryos lack such enrichment.

We next wished to investigate whether random XCI occurred normally in *Eed* ^−*/*−^ embryos. We generated and analyzed postimplantation female embryos from a cross of *Eed^+/−^* animals where the sire also harbored on its X-chromosome both the *GFP* transgene and a mutation in the *Tsix* gene *(Tsix^AA2^* ^Δ*1.7*^ *)* [35]. *Tsix* is a noncoding RNA expressed from the *Xist* locus but in the opposite orientation to *Xist* [35–37]. Whereas *Xist* is required for XCI, intact *Tsix* is necessary to escape XCI. A mutant *Tsix* affects the choice of which X-chromosome is inactivated; the chromosome harboring a mutation in *Tsix* is preferentially rendered inactive [35,38,39]. When inherited on the Xp, the *Tsix* mutation does not alter imprinted XCI (of the Xp). Thus, XCI in preimplantation embryos and extraembryonic lineages, examined above, is unaffected. The epiblast, which normally undergoes random XCI, however, should also now inactivate the Xp exclusively. WT female embryos inheriting the *Tsix^AA2Δ1.7^* allele and the *GFP* transgene on the Xp would therefore be expected to lack GFP expression in their epiblast or epiblast-derived tissues.

We found a gradual decrease in the numbers of green fluorescent protein (GFP)-expressing cells in the epiblast or epiblast derivatives of both WT and *Eed* ^−*/*−^ postimplantation stage embryos that harbored the *Tsix* mutation and the *GFP* transgene on their paternal X-chromosome (*XX^GFP;^* ^Δ*Tsix*^ and *XX^GFP;^* ^Δ*Tsix*^; *Eed* ^−*/*−^, respectively) (Figures 4 and 6). By E8.5, the epiblast-derived tissues of both WT and *Eed* ^−*/*−^ embryos are completely devoid of GFP expression, indicating that XCI is complete and that any residual GFP protein and RNA have either been diluted or degraded by this stage (Figure 6). The lack of GFP-expressing epiblast-derived cells in E8.5 *XX^GFP;^* ^Δ*Tsix*^; *Eed* ^−*/*−^ embryos, thus, indicates that XCI in the epiblast initiates and is stably maintained in the absence of EED.

**Figure 6:**
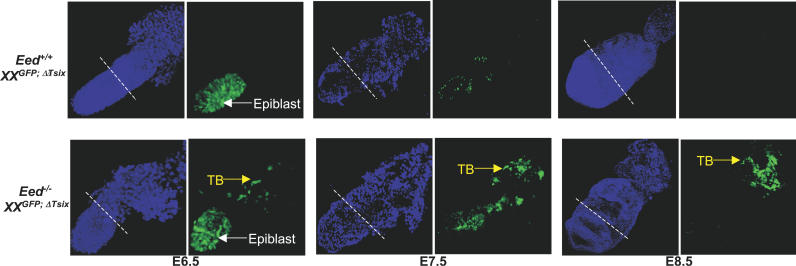
Lack of Defects in Random XCI in Post–Implantation Stage *Eed* ^−*/*−^ Embryos Both WT and *Eed* ^−*/*−^ embryos contain on their Xp a *GFP* transgene and a mutation in the *Tsix* gene (*XX^GFP;^* ^Δ*Tsix*^ and *XX^GFP;^* ^Δ*Tsix*^; *Eed* ^−*/*−^, respectively). The extraembryonic ectoderm (ExE) and its derivatives maintain imprinted XCI of the Xp that is established in all cells of the preimplantation embryo, while the embryonic epiblast lineage undergoes erasure of the imprint at the late blastocyst stage resulting in reactivation of the Xp. This is then followed by random inactivation of either the paternal or maternal X-chromosome during postimplantation development. The *Tsix* mutation on the paternal X-chromosome results in its preferential inactivation in the epiblast. GFP fluorescence in the epiblast or epiblast derivatives of E6.5 and E7.5 embryos is due to residual Xp activity after its reactivation at the late blastocyst stage. By E8.5 when random XCI is complete, epiblast derivatives of both *XX^GFP;^* ^Δ*Tsix*^ and *XX^GFP;^* ^Δ*Tsix*^; *Eed* ^−*/*−^ embryos lack expression of the *Xp-GFP* transgene, indicating that *Eed* is not required for the maintenance of random XCI. Dashed lines delineate border between embryonic and extraembryonic compartments of the embryo; TB, trophoblast cells. *XX^GFP;^* ^Δ*Tsix*^ embryos exhibit a complete absence of *Xp-GFP* fluorescence in the extraembryonic compartment. *XX^GFP;^* ^Δ*Tsix*^; *Eed* ^−*/*−^ embryos, however, harbor increasing numbers of GFP-expressing trophoblast cells, indicating that *Tsix* is not required for reactivation of the Xp.

We confirmed absence of random XCI defects in *Eed* ^−*/*−^ embryos that did not carry the *X^ΔTsix^* allele. We assayed expression of three endogenous X-linked genes, *Hprt, Mecp2,* and *Pgk1,* by fluorescence in situ hybridization (FISH). If random XCI is defective, then both alleles of the X-linked genes should be transcriptionally active. Whereas monoallelic transcription is detected as a single pinpoint in the nucleus, biallelic transcription would be detected as two distinct pinpoints by RNA FISH. We tested female WT and *Eed* ^−*/*−^ E7.5 epiblast derivatives and found that an equal percentage of cells of both genotypes expressed the X-linked genes from only one allele (>96%; Figure 7). These data are further evidence that *Eed* ^−*/*−^ embryos do not suffer defects in random XCI.

**Figure 7:**
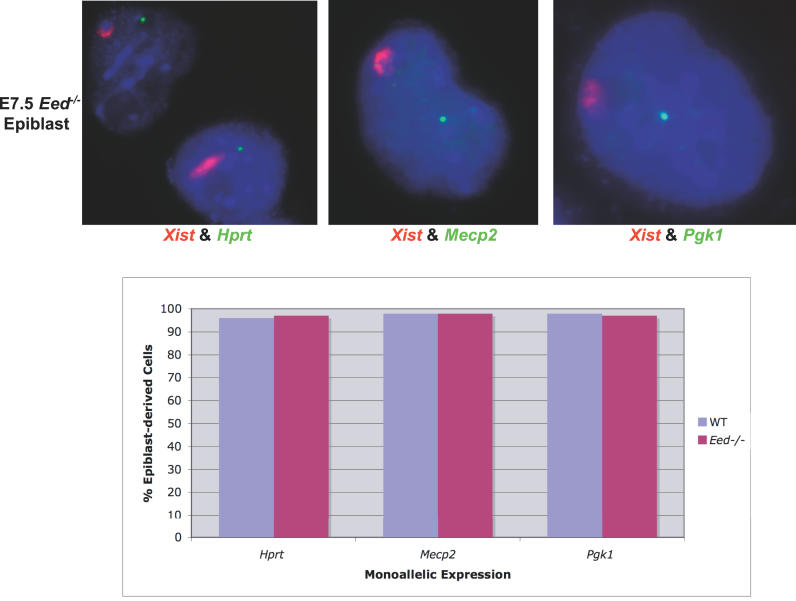
Monoallelic Expression of Endogenous X-linked Genes in Epiblast Derivatives of Female E7.5 *Eed* ^−*/*−^ Embryos FISH detection of *Xist, Hprt, Mecp2,* and *Pgk1* transcripts indicates that endogenous X-linked genes are expressed from one X-chromosome in *Eed* ^−*/*−^ epiblast-derived cells. Epiblast derivatives from both WT and *Eed* ^−*/*−^ E7.5 embryos show similar rates of monoallelic X-linked gene expression (>96%). *Hprt, Mecp2,* and *Pgk1* are invariably expressed from the chromosome other than the one marked by *Xist* RNA coating, which denotes the Xi. Sixty-eight to 87 nuclei were counted for each sample.

Although lacking defects in random XCI, *Eed* ^−*/*−^ embryos are characterized by reactivation of the Xp in differentiating trophoblast cells, as indicated by the increasing numbers of GFP-expressing cells in the ectoplacental cone of *XX^GFP;^* ^Δ*Tsix*^; *Eed* ^−*/*−^ embryos (Figure 6, bottom panels; [31]. The presence of GFP-positive trophoblast cells in *XX^GFP;^* ^Δ*Tsix*^; *Eed* ^−*/*−^ embryos indicates that *Tsix* is dispensable for reactivation of the Xp seen in *Eed*-mutant embryos.

It has been suggested that *Tsix* is required in *cis* for the erasure of the imprint that results in reactivation of the Xp at the late blastocyst stage [40]. The prediction, therefore, would be that a *Tsix* mutation would render the Xp resistant to imprint erasure. However, the presence of GFP-expressing cells in the epiblast of peri-implantation and postimplantation embryos is evidence that the Xp becomes reactivated despite lacking *Tsix* (Figures 3, 4, and 6). *Tsix* may, therefore, also be dispensable for Xp imprint erasure.

## Discussion

Histone methylation by PcGs is among the earliest epigenetic modifications that appear on the Xi during both imprinted and random XCI [18,19,24,25]. It has therefore been proposed that PcGs help initiate XCI [24,25]. Loss-of-function studies in *Eed* ^−*/*−^ embryos have thus far revealed a role for PcGs only in the maintenance of imprinted XCI [30,31]. A defect in initiation of either imprinted or random XCI has not been described in *Eed* ^−*/*−^ embryos [30]. This may be due to the pool of maternally derived Eed protein in the early embryo that lasts long enough to initiate both imprinted and random XCI [18,24,25,32,33].

By analyzing the distribution of EED and/or the histone modification it mediates, H3-3mK27, in preimplantation, peri-implantation, and postimplantation mouse embryos generated from an *Eed^+/^* ^−^ intercross, we have found that maternal EED protein as well as its activity are depleted in *Eed* ^−*/*−^ embryos prior to the initiation of random XCI. We have also demonstrated that postimplantation *Eed* ^−*/*−^ embryos participate in random XCI and do not harbor any random XCI defects, despite the absence of EED protein. Taken together, these results indicate that EED and the histone modification catalyzed by the EED-containing PRC2 complex, H3-3mK27, are dispensable for both the initiation and maintenance of random XCI.

Previously, we reported a strategy of counting and comparing the percentage of cells expressing the paternal X-linked *GFP* transgene versus those that did not in the epiblast of WT and *Eed* ^−*/*−^ embryos, to determine whether *Eed* ^−*/*−^ embryos had a defect in random XCI [30]. An XCI defect would be expected to result in an increased number of GFP-expressing cells in the epiblast lineage of mutant embryos. A dramatic difference in the ratio of GFP+:GFP− epiblast cells between WT and *Eed* ^−*/*−^ embryos was not observed [30]. However, if a relatively small percentage of cells showed expression from both Xs, as is in fact the case in the extraembryonic region of mutant embryos, a defect in random XCI may have been missed using this approach [24,31].

In the current study, we have applied two independent and more sensitive assays to investigate whether random XCI is perturbed in *Eed* ^−*/*−^ embryos. One assay relies on the epiblast expression of the *GFP* transgene from the paternal X-chromosome that also harbors a mutation in the *Tsix* gene. The *Tsix* mutation results in preferential inactivation in the epiblast lineage of the X-chromosome harboring the mutant allele, in this case the Xp and therefore the *GFP* transgene. By biasing random XCI such that the Xp is inactivated, GFP expression in the epiblast lineage would be evidence of a defect in random XCI. The sensitivity of this assay would allow detection of even a few cells that escaped inactivation. As indicated by the lack of GFP expression, by E8.5 both WT and *Eed* ^−*/*−^ embryos achieved complete inactivation of the Xp in every cell of epiblast-derived tissues.

A second assay examines relief from random XCI in *Eed* ^−*/*−^ embryos by testing whether three endogenous X-linked genes are expressed from one or both X-chromosomes in epiblast derivatives by FISH. All three genes show monoallelism in a vast majority of *Eed* ^−*/*−^ cells, equal to that seen in WT epiblast derivatives. The results from both sets of experiments provide strong evidence that random XCI is unaffected in *Eed* ^−*/*−^ embryos.

Since an intact *Tsix* is required for that chromosome to escape XCI and be chosen as the active X, it can be extrapolated that without *Tsix* Xi reactivation would not occur in *XX^GFP,^* ^Δ*Tsix*^; *Eed* ^−*/*−^ embryos. However, the occurrence of imprint erasure in the ICM cells and the presence of GFP-expressing trophoblast cells in the ectoplacental cone of *XX^GFP,^* ^Δ*Tsix*^; *Eed* ^−*/*−^ embryos indicate that the absence of *Tsix* does not prevent Xi reactivation. This is consistent with *Tsix* being upstream of the epigenetic modifications that characterize the Xi. *Tsix* transcription inhibits *Xist* transcription, which in turn is thought to recruit the silencing machinery, i.e., PcGs, to the prospective Xi [24,35,39,41,42]. In this cascade, therefore, factors that mediate transcriptional silencing act downstream of *Tsix*. If one of these downstream components is missing, XCI may be defective (i.e., in *Eed* ^−*/*−^ embryos) or reversed (i.e., during Xp imprint erasure) even if the antisilencing factor *Tsix* is lacking.

In in vitro assays, the PcG PRC1 complex can compact nucleosomes and repress transcription [12,13]. We have shown here that the Xi-enriched EED, whose activity is required for H3-3mK27, a prerequisite for PRC1 function, is not necessary for the formation of the Xi-heterochromatin and the accompanying transcriptional silencing. The EED-containing PRC2 complex appears to be required in XCI only to prevent reactivation of the imprinted Xi during differentiation of the trophectoderm lineage [31]. In the epiblast, PcGs may in fact play a similar role. However, an XCI defect in this lineage may not be manifested in *Eed* ^−*/*−^ embryos until after their death at E9.5. Alternatively, epigenetic modifications that play a prominent role in random XCI, such as cytosine-guanine dinucleotide DNA methylation, may compensate for the absence of EED in the epiblast. In support of this idea, Sado et al. [43] have shown that a mutation in the *Dnmt1* gene does not affect imprinted XCI, but does perturb random XCI; epiblast-derived cells in *Dnmt1* mutant embryos appear to harbor two active X-chromosomes.

## Materials and Methods

### Mice.

The *Eed^17Rn5-3354SB^* line of mice originated in a mutagenesis screen [44] and have been maintained in heterozygosity and genotyped as previously described [30,32]. The *X^GFP^* is the D4/XEGFP transgenic line [34]. Mice harboring the *Tsix^AA2^* ^Δ*1.7*^ mutation, which results in an absence of mature *Tsix* RNA and thus is a null allele, were generated from targeted ES cells that were a kind gift of Takashi Sado and have been described elsewhere [35].

### Embryo dissections.

Isolation of E3.5-E7.5 embryos was performed using established procedures [45]. E4.5 embryos were collected similarly to E3.5 embryos. All embryos were flushed or dissected in DMEM or PBS with 5% fetal calf serum, to prevent their sticking to glass pipets. Reichert's membranes were removed using forceps from all postimplantation embryos prior to IF stainings and confocal imaging.

### IF.

Antibody stainings of intact embryos were performed essentially as described [24,46]. The embryos were fixed overnight in 4% paraformaldehyde at 4° C. After three washes in PBS, the embryos were permeabilized with 0.5% Triton X-100 for 20 to 30 min. The embryos were then blocked with 0.25% fish skin gelatin (stock is 45%; catalog No. 7765; Sigma, St. Louis, Missouri, United States). The primary antibodies incubations were carried out at 4° C overnight in blocking solution with 5% normal goat serum. The Eed and H3-3mK27 antibodies have been described previously and were used at 1:50 and 1:200 dilutions, respectively [24,47]. Washes were then done with 0.25% fish skin gelatin and PBS (three times, 5 min each). Secondary antibodies used were coupled to Alexa 594 (Molecular Probes, Eugene, Oregon, United States) or Cy5 (Amersham, Piscataway, New Jersey, United States). The embryos were washed in PBS and mounted in thin depression glass slides (catalog No. 12–560; Fisher Scientific) with Vectashield with DAPI (Vector Labs, Burlingame, California, United States).

### FISH.

RNA FISH was carried out as described [31].

### IF-FISH.

E4.5 embryos and dissociated epiblast cells from E6.5 embryos that were processed for both IF and FISH underwent IF before FISH. E4.5 embryos were flushed and cytospun (Shandon Cytospin 3; Shandon, Waltham, Massachusetts, United States) onto gelatin-coated glass slides in 200 μl of PBS with 10 mg/ml BSA (NEB, Ipswich, Massachusetts, United States) and 150 units/ml RNasin RNase inhibitor (Promega, Madison, Wisconsin, United States) (PBS/BSA/RNasin) at 1,000 rpm for 6 min. The site of embryos was encircled with a Pap Pen. After brief drying (3 to 5 min), the samples were fixed in 4% paraformaldehyde for 10 min in a humid chamber. After brief washes in PBS, the embryos were permeabilized with 0.5% Triton X-100 in PBS containing 10 μl/ml ribonucleoside-vanadyl complex (NEB) for 7 min. Following brief washes in PBS, the embryos were blocked with PBS/BSA/RNasin containing 0.2% Tween-20 for 20 to 30 min at room temperature. The samples were then incubated in primary antibodies diluted in blocking buffer for 1 h at 37° C. After brief washes in PBS, the embryos were incubated in secondary antibodies diluted in blocking buffer for 45 min at 37° C. After washes in PBS, the embryos were postfixed in 2% paraformaldehyde. Following brief washes in PBS, the samples were dehydrated by sequential incubations in 70%, 85%, 95%, and 100% ethanol, for 2 min each. The embryos were then air-dried and processed for FISH. Epiblast and epiblast-derived cells from E6.5 and E7.5 embryos were dissociated and cytospun as described [31]. IF and/or FISH was then carried out as above. The *X-GFP* transgene facilitated identification of epiblast and its derivatives during dissociation of the embryo.

### Microscopy.

All images of embryos (Figures 1–4 and 6) are a composite of entire set of confocal sections that were obtained using a Leica SP2 confocal microscope and processed using the LeicaLCS software. All other images of IF and FISH stains (Figures 5 and 7) were acquired using a Leica DML fluorescence microscope and SPOT RT software. Additional details are provided elsewhere [31].

## Abbreviations

E: embryonic day
FISH: fluorescence in situ hybridization
GFP: green fluorescent protein
H3-K27: lysine 27 of histone H3
H3-3mK27: histone H3 trimethyl lysine 27
ICM: inner cell mass
IF: immunofluorescence
Pc: Polycomb
PcG: Polycomb group
PRC: Polycomb repressive complex
WT: wild-type
XCI: X-chromosome inactivation
Xi: inactive X-chromosome
Xp: paternal X-chromosome
